# A multicentre randomised controlled trial to evaluate the efficacy, morbidity and functional outcome of endoscopic transanal proctectomy versus laparoscopic proctectomy for low-lying rectal cancer (ETAP-GRECCAR 11 TRIAL): rationale and design

**DOI:** 10.1186/s12885-017-3200-1

**Published:** 2017-04-11

**Authors:** Bernard Lelong, Cécile de Chaisemartin, Helene Meillat, Sandra Cournier, Jean Marie Boher, Dominique Genre, Mehdi Karoui, Jean Jacques Tuech, Jean Robert Delpero, the French Research Group of Rectal Cancer Surgery (GRECCAR)

**Affiliations:** 1 Department of Digestive Surgical Oncology, Department of Mini Invasive Interventions (DIMI), Paoli Calmettes Institute, Comprehensive Cancer Centre, Marseille, France; 2 Department of Clinical Research and Innovation (DRCI), Paoli Calmettes Institute, Comprehensive Cancer Centre, Marseille, France; 3 Department of Biostatistics and Methodology, Paoli Calmettes Institute, Comprehensive Cancer Centre, Marseille, France; 4 Department of Digestive Surgery, CHU Pitié-Salpetriere, Paris, France; 5 Department of Digestive Surgery, CHU Charles Nicolle, Rouen, France

**Keywords:** Rectal cancer, Minimally invasive, Transanal approach

## Abstract

**Background:**

Total mesorectal excision is the standard surgical treatment for mid- and low-rectal cancer. Laparoscopy represents a clear leap forward in the management of rectal cancer patients, offering significant improvements in post-operative measures such as pain, first bowel movement, and hospital length of stay. However, there are still some limits to its applications, especially in difficult cases. Such cases may entail either conversion to an open procedure or positive resection margins. Transanal endoscopic proctectomy (ETAP) was recently described and could address the difficulties of approaching the lower third of the rectum. Early series and case-control studies have shown favourable short-term results, such as a low conversion rate, reduced hospital length of stay and oncological outcomes comparable to laparoscopic surgery. The aim of the proposed study is to compare the rate of positive resection margins (R1 resection) with ETAP versus laparoscopic proctectomy (LAP), with patients randomly assigned to each arm.

**Methods/design:**

The proposed study is a multicentre randomised trial using two parallel groups to compare ETAP and LAP. Patients with T3 lower-third rectal adenocarcinomas for whom conservative surgery with manual coloanal anastomosis is planned will be recruited. Randomisation will be performed immediately prior to surgery after ensuring that the patient meets the inclusion criteria and completing the baseline functional and quality of life tests. The study is designed as a non-inferiority trial with a main criterion of R0/R1 resection. Secondary endpoints will include the conversion rate, the minimal invasiveness of the abdominal approach, postoperative morbidity, the length of hospital stay, mesorectal macroscopic assessment, functional urologic and sexual results, faecal continence, global quality of life, stoma-free survival, and disease-free survival at 3 years. The inclusion period will be 3 years, and every patient will be followed for 3 years. The number of patients needed is 226.

**Discussion:**

There is a strong need for optimal evaluation of the ETAP because of substancial changes in the operative technique. Assessment of oncological safety and septic risk, as well as digestive and urological functional results, is particularily mandatory. Moreover, benefits of the ETAP technique could be demonstrated  in post-operative outcome.

**Trial registration:**

ClinicalTrial.gov: NCT02584985.

**Date and version identifier**: Version n°2 – 2015 July 6.

## Background

### Total mesorectal excision: the surgical gold standard for mid- to low-rectal cancer

The standard surgical treatment for mid- and low-rectal cancer is total mesorectal excision (TME). Originally performed with open surgery, TME demonstrated improved local control and reduced urogenital morbidity compared with the non-standardized procedure [1, 2]. The circumferential resection margin (CRM) is the strongest independent factor for both survival and local control [3, 4]. The TME technique has also been shown to be diffusible and reproducible with adequate educational programmes [5].

### Laparoscopic approach: improved short-term outcomes but residual limitations

The laparoscopic approach has been validated by several randomised controlled trials in the previous decade. The laparoscopic approach offers the patient better post-operative recovery, a lower risk of wound hernia and oncological results comparable to those of open surgery [6–8]. However, the risk of conversion to an open procedure remains significant (from 7 to 34%) [9–11], especially in difficult situations, such as patients with cancer in lower third of the rectum, male patients with a narrow pelvis, and patients with obesity [9, 12]. Recently, two randomised trials showed unsatisfactory results for the laparoscopic approach compared with laparotomy: post-operative short-term outcomes were comparable, and the oncological quality of the resection was better in the open surgery group; however, the methodology used in these studies (composite criterion) and the surgical technique (hybrid technique) were questionable [13, 14]. On the other hand, a robotic approach offers optimised vision and manipulation, which could provide an advantage in terms of pelvic nerve preservation [15], although recent randomised trials have failed to demonstrate improved short-term outcomes.

### Endoscopic transanal proctectomy (ETAP): rationale and current evaluation

ETAP allows retrograde mesorectal excision in which the whole pelvic dissection can be performed via a specific, moderate-cost transanal device. The procedure is then completed with a shorter transabdominal laparoscopic step in which the colon is mobilised and inferior mesenteric vessel ligation is performed prior to low coloanal anastomosis. The originality of this approach is that it allows TME without peritoneal and abdominal wound trauma. This represents a new technical improvement in the area of minimally invasive pelviabdominal surgery that uses a natural orifice for surgical access. This approach offers a closer view and better exposure of the pelvic dissection plane and could therefore improve oncological quality and pelvic nerve preservation [16, 17]. It could be beneficial to postoperative patient outcomes.

This technique has been shown to be feasible and reproducible in previous clinical series [18–20]. Its conversion rates appear to be lower than the published rates for a laparoscopic approach at markedly less than 10%. The compiled rates of morbidity, R1 resection, and mesorectal macroscopic integrity appeared to be comparable to the results for the laparoscopic approach. However, functional results and long-term survival need to be evaluated in comparative studies. According to some authors, the benefit of the transanal approach is significant in difficult cases, such as cases of male patients with a narrow pelvis [21]. More recently, larger single- and multicentre series confirmed the oncological safety and surgical security of these promising techniques [22–25]. At the same time, however, some papers have described specific complications arising from the use of an inadequate technique [26], emphasising the essential need for educational programmes and training. Three recently published non-randomized comparative studies presented conclusions similar to those of our institutional study. While none of these three studies showed any oncological superiority, they did report substantial advantages in short-term outcomes with trans-anal endoscopic proctectomy compared with standard laparoscopy [27–29]. Finally, we performed a single-institution case-controlled comparative study (*n* = 72) [30] and found that ETAP could make the procedure easier and improve short-term outcomes without impairing the oncological quality or outcome. Indeed, we observed a lower conversion rate (2.9% vs 23.6%; *p* = 0.011), shorter in-hospital stay (8 vs 9 days; *p* = 0.038), and fewer readmissions (0% vs 15.8%; *p* = 0.03) in the ETAP group. Comparable morbidity rates (27% vs 34%; *p* = 0.52) and functional results (Kirwan score 1/2; 80.3% vs 80.6%; *p* = 0.94) [31] were also found. In parallel, the oncological quality criteria were comparable (R1 resection 5.9% vs 10.5%; *p* = 0.74; grade 3 mesorectal integrity 57.5 vs 56.2; *p* = 0.99).

There was a clear expected benefit for the patients who underwent the ETAP procedure in terms of postoperative short-term outcomes, the risk of conversion to an open procedure, and the risk of wound hernia. This trial also suggested potential significant advantages in terms of dissection quality, specimen quality, and nerve preservation quality.

A well-designed multicentre RCT with a large sample of patients is the best option for obtaining clinical evidence to support the use of a novel surgical technology.

The internal validity of the surgical technique among the surgeons participating in this RCT is a crucial prerequisite for this surgical RCT. To date, no potential learning process for transanal endoscopic proctectomy has been assessed. This could be significant because this new “bottom-up” approach has several intricacies that have been well described by several authors. In a previous study, we showed that the learning process for laparoscopic TME affected post-operative morbidity for the first fifty patients [32]. We can therefore presume that the learning process remains significant, even in teams highly experienced in laparoscopic colorectal resections. Several previous trials were affected by the learning process, likely because of a lack of selection and/or formation of the participating teams [9]. In contrast, educational programmes in rectal cancer surgery have already been shown to be efficient [5]. Thus, it seemed essential to build an educational programme dedicated to the transanal endoscopic proctectomy technique associated with the study project. To this end, an educational committee of three expert surgeons was created to validate the participating teams.

Below, we propose, with the support of the GRECCAR (French Research Group of Rectal Cancer Surgery), the protocol for the ETAP-GRECCAR 11 RCT to compare endoscopic transanal proctectomy with laparoscopic proctectomy for low-lying rectal cancer.

## Methods / design

*Objectives*: The purpose of the ETAP-GRECCAR 11 RCT is to evaluate, in a randomised trial, the impact of ETAP compared with standard laparoscopic proctectomy for low-lying rectal cancer requiring manual coloanal anastomosis on the rate of resection R1. Secondary outcomes included the conversion rate, the quality of the mesorectal excision, postoperative morbidity, quality of life, stoma-free survival and disease-free survival at 3 years.

### Study design (Fig. 1)

**Fig. 1:**
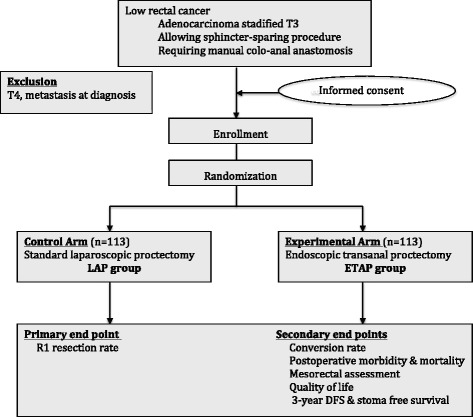
Study scheme of ETAP-GRECCAR 11 trial, inclusion and exclusion criteria, intervention, and end points (DFS, Disease-Free Survival)

This RCT is a national, multicentre, randomised, open-labelled, parallel-assigned, controlled, and non-inferiority trial comparing the oncologic results (R1 resection rate) of ETAP and LAP for low-lying rectal cancer requiring manual coloanal anastomosis. It is schematically described in Fig. 1.

This study was approved by a National Institutional Review Board (*Comité de Protection des Personnes Sud Méditerranée 1, Ref N°1–15 63:08/07/2015)* and by the National Agency of Medicine and Medical Products (ANSM: 150695B-12). This study is supported by a grant from the French Ministry of Health (PHRC-K14-112). All investigators will proceed with this study in accordance with the Declaration of Helsinki. Written informed consent will be obtained from all patients before they are recruited. The trial has been registered in the database of clinical trials (NCT 02584985). This RCT will be monitored by an independent data and safety monitoring committee (DSMC) organised by the Department of Clinical Research and Innovation of Paoli Calmettes Institute.

### Study population and eligibility criteria

The patient inclusion and exclusion criteria are as follows:

- Inclusion criteria
  - Patients aged >18 years with no upper age limit
  - Non-metastatic staged T3 rectal adenocarcinoma allowing a sphincter-sparing procedure
  - Tumour location or local condition justifying manual coloanal anastomosis
  - Patient eligible for surgery
  - Written informed consent
  - Affiliation with the social security system.
- Exclusion criteria
  - A tumour staged as T4 with en-bloc resection
  - Possible mechanical trans-sutural anastomosis
  - Distant metastasis at diagnosis
  - Any psychological, familial, sociological or geographical conditions that could hamper compliance with the study protocol or the follow-up schedule
  - Patients who have been deprived of their liberty or placed under the authority of a legal guardian.

### Participants—educational programme and committee

The institutional promoter is the Paoli Calmettes Institute Department of Clinical Research and Innovation (DCRI). Patients are included from several units of colorectal surgery in France (see list of participating centers in the Ackowledgments section; also see below the team selection and teaching programme information). The study has been approved by the scientific board of the GRECCAR group. This group was created by surgical teams in France who are involved in the management of rectal cancer with the aim of conducting and publishing multicentre clinical trials in high level journals on the subject, and expanding this surgical specialty to various learned societies. Most of the participating teams in the study are affiliated with the GRECCAR group.

To participate in this RCT, preliminary experience with 50 laparoscopic recto-sigmoidal resections and preliminary experience with 10 endoscopic transanal proctectomies were required. An educational committee of three expert surgeons (CDC, JJT, MK) was established to assess technical skills by viewing videos submitted by the team and approve teams for participation.

To ensure the formation of colorectal teams with insufficient experience and to allow for their secondary participation in the trial, two experimental workshops have been planned for the year 2016 (March and October). European experts will participate in the workshops, which will include animal and cadaver dissection. After this first step, the learning team will begin to perform procedures at their home hospitals with the assistance of a visiting expert surgeon.

Finally, clinical workshops that include live surgery and expert presentations have been held annually by the coordinating team in Marseille since 2015 to promote the standardisation of the operative technique through discussions among the teams.

### Randomisation

After completion of the pre-intervention assessments, which will include baseline functional and quality of life assessments, the patients will be randomised and assigned to a surgical approach (ratio 1:1). Blocked centralised randomisation with stratification by centre will be prepared by the DCRI of Paoli Calmettes Institute.

### Surgical procedure

#### Experimental arm: ETAP

For the primary transanal approach, the patient is careful positioned in lithotomy, and the anorectal junction is exposed with a standard retractor. Endoanal dissection includes mucosal incision and internal sphincter dissection according to the tumour extension; i.e., mucosal excision or partial or total internal sphincter resection (ISR), as described by Rullier et al. [33]. Primary conventional dissection up to the circumferential exposure of the fascia recti is recommended prior to endoscopic dissection. The transanal endoscopic device can be then positioned to perform the whole mesorectal excision. Mesorectal endoscopic dissection will then be performed according to the following procedures:

1. posterior dissection up to the vertical segment of the rectum
2. anterior dissection with final opening of the Douglas pouch
3. lateral dissection with nerve-sparing dissection.

ETAP is considered complete if all 3 steps are achieved.

In the secondary transabdominal approach, laparoscopy with multiple ports or a single-port will then be performed for colonic mobilisation and vascular ligation. The specimen will be extracted through an abdominal wound or transanally. A coloanal manual anastomosis will then be performed. A systematic loop ileostomy/colostomy is mandatory.

#### Control arm: LAP

A primary transanal conventional dissection will be performed to assess sphincter preservation. A standard multiport or single port laparoscopic approach will be performed for colonic mobilisation, vascular ligation, and anterograde total mesorectal excision, with a nerve-sparing dissection. The specimen will be extracted through an abdominal wound or transanally. A coloanal manual anastomosis will then be performed. A systematic loop ileostomy/colostomy is mandatory.

### Outcome and assessments

#### Primary outcome

The primary outcome is the R1 resection rate, defined as a circumferential resection margin (CRM) ≤ 1 mm, and/or a distal positive margin.

#### Secondary outcomes

The secondary outcomes are as follows:

- Conversion rate
- Single or multiport abdominal surgery
- 90-day postoperative morbidity
- Length of hospital stay
- Mesorectal macroscopic assessment
- Functional and quality of life assessments
- Stoma-free survival at 3 years
- Local control and disease-free survival at 3 years.

### Data registration

#### Surgical data

During surgery, the operating data will be recorded on the electronic case report form (e-CRF), which includes the operative time for each step, intraoperative difficulties and conversion, an assessment of nerve preservation, and the completion of the ETAP according to the criteria defined in the Methods section.

Will be considered as a conversion, the need for laparotomy in both groups; but also the use of endoscopic transanal access platform in patients in the control group.

#### Morbidity and mortality

Postoperative complications will be noted by the surgeon in the e-CRF during hospitalisation and during the first 3 months postoperatively. Postoperative death is defined as death occurring within 30 postoperative days or during the first hospitalisation. Postoperative complications are defined by any deviation from the normal post-operative course within 90 postoperative days or during the first hospitalisation. Morbidity will be evaluated according to the Clavien-Dindo classification of surgical complications [34].

#### Pathological data

Distal and circumferential margins will be scrupulously recorded as components of the primary outcome measures. Particular attention will also be paid to the macroscopic mesorectal assessment, as defined by Quirke et al. [35], as an essential surgical quality criterion. The number of resected and invaded nodes, tumour differentiation, the presence of vascular embolisms (venous or lymphatic, intra or extra-mural), and perineural invasion will also be assessed. The resected specimens will be staged according to the American Joint Committee on Cancer (AJCC) criteria, 7th version [36].

#### Functional and quality of life assessments (Table 1)

**Table 1:** Summary of the follow-up visit schedule and assessed parameters at each time point. (CEA, carcinoembryonic antigen; MRI, Magnetic Resonance Imaging; CT, computed tomography; QoL, Quality of life)

ETAP follow-up schedule	Inclusion	Surgery	3 months	6 months	12 months	24 months	36 months
Clinical evaluation	X	X	X	X	X	X	X
Surgical complications		X	X				
CEA	X		X	X	X	X	X
Endorectal utrasound	X						X
Rectal MRI	X						X
CT thorax/abdomen or TEP-FDG	X			X	X	X	X
QoL	X		X	X	X	X	X

To ensure the exhaustiveness of the data, baseline functional and quality of life assessments will be required before randomisation. The functional scales used will include the Urinary Symptom Profile scale (USP) for urinary function [37] and the FSFI [38] scale for female patients and the simplified IIEF-5 [39] for male patients for sexual function. Faecal continence will be evaluated with the Cleveland score [40]. Global quality of life will be assessed with the EORTC QLQ-C30 scale [41]. The patients in both arms will be evaluated preoperatively and at months 3, 6, 12, 24 and 36. It is schematically described in Table 1.

#### Oncological follow-up (Table 1)

Oncological status (no disease or local or metastatic recurrence) and stoma status (stoma-free, persistent or re-do) will be determined at the same intervals. Typical follow-up will include a clinical exam, CEA analysis, CT scan or chest radiography with abdominal ultra-sound at months 3, 6, 12, 24 and 36.

### Sample size and statistical considerations

#### Statistical methodology

The trial is designed as a non-inferiority trial. In statistical terms, the study will establish whether the R1 resection rate (%) for the experimental treatment is not worse than the R1 resection rate (%) for the standard treatment by more than a specific non-inferiority margin, δ_0_ set to 0.05, with δ denoting the difference in R1 resection rates (experimental vs control). Formally, the study will test the one-sided null hypothesis H0: δ > = 5% vs the alternative H1: δ < 5%, and will reject the null hypothesis with a 5% level of significance if the upper bound of a standard asymptotic 90% confidence interval for difference in proportions is below the 5% non-inferiority margin.

#### Sample size

In a recent retrospective study (*n* = 72) that compared oncological quality criteria, the R1 resection rates (%) in the ETAP and standard LAP groups were 5.9 and 10.5%, respectively [30].

We therefore hypothesised a R1 resection rate (%) of 10% in the control arm and a 4% reduction in the experimental arm over the standard arm (6% vs 10%).

Based on the non-inferiority hypothesis and the above findings, a total of 226 patients (113 for each arm) is required to accept a difference in the R1 resection rates (experimental vs control) that is no worse than 5% with 80% power and a 5% error risk.

The inclusion period will be 3 years, and the study will run for 6 years. The short-term results will be presented before the end of 2018. Analyses of survival and functional outcomes will be performed after at least 3 years of follow-up, and the results will be available in 2020.

## Discussion

The ETAP trial is the first randomised trial with an active inclusion process to compare ETAP with a standard transabdominal laparoscopic approach. The first patient, n°01001, was included on January 26, 2016. In this non-inferiority trial, we hypothesised that ETAP would have similar oncologic results (R1 resection rate) with significant improvements in the quality of dissection (mesorectal assessment and nerve preservation), morbidity, function and quality of life compared with laparoscopic proctectomy.

Some may question the need for randomisation to evaluate ETAP because it is “just a U-turn in the approach to the same operation”. We strongly believe that an optimal evaluation that includes randomisation is mandatory for several reasons.

### Assessment of oncological safety and septic risk

ETAP is initiated from the endoluminal space to the extraluminal space, which is the opposite of the classic top-to-bottom approach of TME. Under these conditions, there is a theoretical risk of bacterial contamination and tumour spillage, even with meticulous closure of the rectal lumen after endoanal dissection. Volthuis et al. recently described the correlation between bacterial contamination (positive culture) and septic pelvic complications [42], reporting an alarming rate of 39% of patients with a positive culture and 17.4% of patients with septic complications (nearly half of the patients with a positive culture).

Regarding the theoretical risk of tumour spillage, there are few data at present on mid-term oncological follow-up, and no alarming rates have been reported. Lacy et al. recently reported a 90.8% disease-free survival rate and 0.8% local recurrence rate in a large series of 140 patients, but their study had a limited follow-up of a median of 15 months [22]. Another study published by Tuech et al. reported the outcome of 56 patients with a median of 29 months’ follow-up . The 5-year estimated disease-free survival was 94.2%, and the local recurrence rate was 1.9% [24]. However, only a randomised evaluation can assess the oncological safety of this promising technique. The ETAP-GRECCAR 11 trial will assess and compare the overall local control and disease-free survival at 36 months in both groups.

### Assessment of functional safety

#### Faecal continence

A transanal primary conventional approach and the positioning of the endoscopic device in the anal canal could affect sphincter function. A conservative procedure for low rectal cancer with coloanal anastomosis can result in frequent faecal continence dysfunction [43]. In this domain, a new technique that includes a theoretical risk of incontinency must be evaluated carefully. To date, few specific data are available; e.g., in Tuech et al. [24], the median Wexner score at 1 year was 5 (range 3–18) and three patients required a secondary stoma (7.3%). These results are consistent with the current literature on coloanal anastomosis. Anal function has been evaluated after TEM; e.g., manometric analyses of the effects of anal dilatation after TEM indicated a decrease in sphincter tonus ranging from 2.5 to 37% compared with preoperative sphincter pressure, with complete recovery to clinical continence 6 to 16 weeks postoperatively [44]. However, prolonged anal dilatation during ETAP (100 min as a median in the study of Tuech et al. [24]) could induce more sphincter function problems. Therefore, the ETAP trial will assess and compare the functional Wexner scores of both arms.

### Pelvic nerve preservation and urogenital function

ETAP allows mesorectal excision from the transanal endoscopic platform with magnified vision and exposure. Therefore, we can hypothesise that the quality of exposure would favour pelvic nerve preservation. However, some anatomic studies have noted specific risks associated with this bottom-up approach. In particular, dissecting too laterally along the pelvic fascia can result in lateral nervous plexus injury. Thus, it is recommended to lead the dissection from a posterior to a lateral position and from an anterior to a lateral position. In this manner, adequate identification of the neurovascular bundle is possible when a transanal approach is used [17, 26].

Again, current clinical series offer a limited evaluation of pelvic nerve preservation with ETAP. In a comparative study, Fernández-Hevia et al. reported a non-significant change (from 11 to 3%) in the urinary retention rate for the ETAP group [28].

Moreover, no existing study has reported sexual function outcomes. The ETAP-GRECCAR 11 trial will assess and compare urinary and sexual function and specific quality of life for both arms of the study.

## Conclusions

The proposed ETAP-GRECCAR 11 trial represents a multicentre randomised controlled trial to demonstrate the oncological safety and improved postoperative morbidity and quality of life following ETAP for low rectal cancer compared with the standard LAP. We believe that this trial will significantly contribute to the evolution of surgical practice in low rectal cancer.
